# Comparative Action of Blue Food Colorants (Genipin, Patent Blue V, and Brilliant Blue FCF); Their Effect on Oxidative Stress in Human Plasma and Blood Platelets In Vitro

**DOI:** 10.3390/ijms27136045

**Published:** 2026-07-06

**Authors:** Beata Olas, Bogdan Kontek, Dagmara Witkowska, Karolina Sitek

**Affiliations:** Department of General Biochemistry, Faculty of Biology and Environmental Protection, University of Lodz, 90-236 Lodz, Poland; bogdan.kontek@biol.uni.lodz.pl (B.K.); dagmara.witkowska@edu.uni.lodz.pl (D.W.); karolina.sitek@edu.uni.lodz.pl (K.S.)

**Keywords:** blood platelets, blue food colorant, genipin, oxidative stress, plasma

## Abstract

The influence of natural and synthetic blue food colorants on the human body, including the cardiovascular system, is a complex and not fully understood topic. Considering that various papers have demonstrated that oxidative stress is a crucial step in the development of cardiovascular diseases (CVDs), our experiments on the pro- or antioxidant action of three blue food colorants (one natural colorant—genipin—and two synthetic colorants—brilliant blue FCF and patent blue V) focused on two aspects that are important for the development of CVDs: the level of biomarkers of oxidative stress induced by H_2_O_2_/Fe^2+^ (the donor of hydroxyl radicals—one of the most aggressive reactive oxygen species produced in humans) in human blood platelets and human plasma, as well as the arachidonic acid cascade in blood platelets stimulated by thrombin (in vitro). Our results demonstrated that two tested blue colorants—genipin and brilliant blue FCF (at four used concentrations: 2, 10, 20, and 200 µM)—reduced plasma lipid peroxidation induced by H_2_O_2_/Fe^2+^. Moreover, all tested blue colorants (genipin, brilliant blue FCF, and patent blue V; at the concentrations 2, 10, 20, and 200 µM) inhibited lipid peroxidation in blood platelets treated with H_2_O_2_/Fe^2+^. In contrast, only genipin (at the highest used concentration—200 µM) statistically significantly reduced plasma protein carbonylation induced by H_2_O_2_/Fe^2+^ (inhibition of this process: about 25%). However, all tested food colorants decreased blood platelet protein carbonylation stimulated by H_2_O_2_/Fe^2+^, but their action was not always statistically significant. In addition, we noted that all used blue food colorants (1–200 µM) have protector effects on the change in the level of thiol groups in plasma proteins stimulated by H_2_O_2_/Fe^2+^, but these tested colorants change the level of thiol groups in blood platelets treated with H_2_O_2_/Fe^2+^ only at the highest used concentration—200 µM. In conclusion, the present study provides the first data on the antioxidant potential of genipin, brilliant blue FCF, and patent blue V in selected elements of blood treated with H_2_O_2_/Fe^2+^. Earlier and current studies have indicated the promising potential of these blue food colorants, especially genipin (without cytotoxicity toward human blood platelets), which can modify the oxidative stress of platelets and plasma in vitro at concentrations (1–200 µM) which can be obtained in blood during its administration. However, the presented results have limitations, especially concerning the mechanistic clarity surrounding the antioxidant properties of the tested blue food colorants. Therefore, further in vivo experiments are needed to provide a better understanding of their antioxidant potential.

## 1. Introduction

Oxidative stress is a process which may amplify the action of various factors and perpetuate cellular damage. For example, excessive reactive oxygen species (ROS) production leads to protein oxidation, DNA damage, and lipid peroxidation. Moreover, different cells, including activated blood platelets, release additional ROS, aggravating oxidative stress, a hallmark of various diseases, including cardiovascular diseases (CVDs). Blood platelet activation is also associated with arachidonic acid metabolism, in which various intermediate products (for example, thromboxane A_2_) are produced [1,2,3].

Various chemical compounds presented in foods may modulate the level of oxidative stress. However, the influence of natural and synthetic blue colorants on this process in various elements of human blood is not well known. In addition, phytochemicals found in plants have demonstrated significant potential in the treatment and prevention of diseases associated with oxidative stress. From an ethnopharmacological perspective, plants traditionally used to alleviate oxidative stress, such as *Gardenia jasminosis* J. Ellis and *Genipia americana* L., are promising candidates for antioxidant potential. *G. jasminosis* fruits are also used as functional food supplements in East Asia. For example, they are often used as dietary supplements mixed with health foods or tea [4].

The hydrolysis of geniposide and gardenoside by β-glucosidase results in the production of genipin (obtained from *G. jasminosis* and *G. americana*), a water-soluble iridoid monoterpenoid whose maximum absorbance (496 nm) does not change with the pH of the environment. Genipin can be reacted with amino acids such as glycine, lysine or phenylalanine to obtain the blue dye gardenia blue. In addition, plant-derived bioactive compounds, including genipin, offer multi-targeted properties (antibacterial, anti-inflammatory, antithrombotic, antioxidant, and others) and low cytotoxicity. Recent reviews indicate a growing interest in genipin, which acts as a precursor of blue pigments and a natural crosslinking agent for food and biomaterial-related applications [5,6]. For example, genipin is used as blue colorant in dessert, gel, juices, nectars, and beverages. Although genipin-based colorants such as gardenia blue have undergone genotoxicity testing, available toxicological data—especially for various elements of blood (including blood cells)—remain limited and sometimes equivocal [7]. In addition, our preliminary in vitro studies suggest that genipin can modulate hemostatic properties of blood components without overt cytotoxicity toward human platelets [4,7]. However, the effect of genipin on the parameters of oxidative stress in human plasma and blood platelets is not documented. Our study is the first work devoted to a comprehensive assessment of the biological action of genipin, employing an in vitro experimental system related to oxidative stress in human blood platelets and human plasma. Pro- or antioxidant properties of genipin were assessed in human plasma and human blood platelets treated with H_2_O_2_/Fe^2+^ (the donor of hydroxyl radicals—one of the most aggressive ROS produced in human). Additionally, we investigated the effect of genipin on enzymatic lipid peroxidation—arachidonic acid metabolism in blood platelets activated by thrombin (in vitro). The pro- or antioxidant potential of genipin was examined in the concentration range of 1–200 µM, which was chosen based on our previous study [6] and literature data, which indicate that these concentrations can be achieved in vivo with its supplementation [8,9].

**Figure 1:**
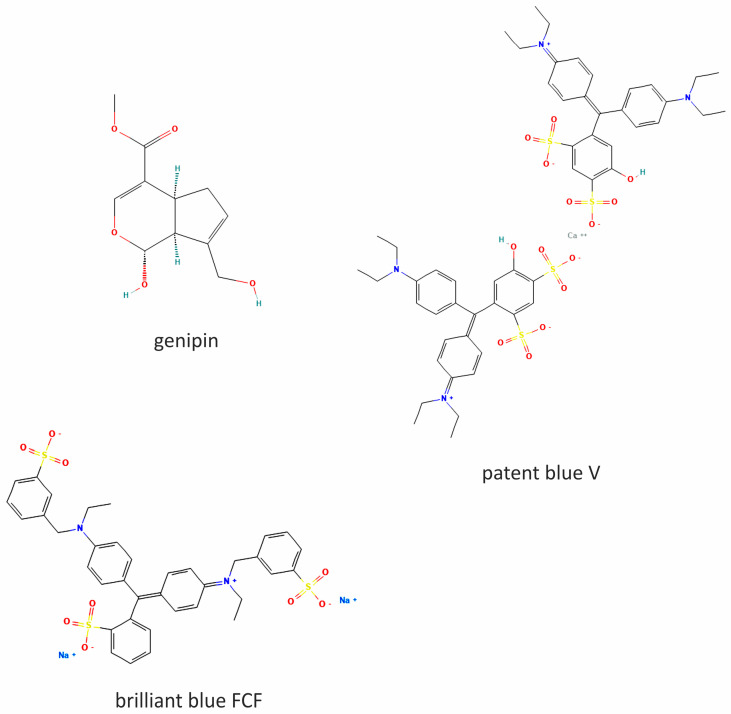
The chemical structures of genipin, patent blue V, and brilliant blue FCF. The 2D structures were obtained from PubChem [accessed on 10 June 2024].

We also compared the biological action of genipin with the properties of two synthetic blue colorants (brilliant blue FCF and patent blue V: 1–200 µM), as our earlier experiments demonstrated that they modulate various steps of blood platelet activation and coagulation times in vitro [7]. Importantly, a concentration of 1 µM can be attained in blood during their administration [8,10]. Ascorbic acid (vitamin C, which is a potent water-soluble antioxidant; 10 µM) was used as a reference antioxidant in our in vitro model. Figure 1 shows the chemical structures of genipin, patent blue V, and brilliant blue FCF.

## 2. Results

**Figure 2:**
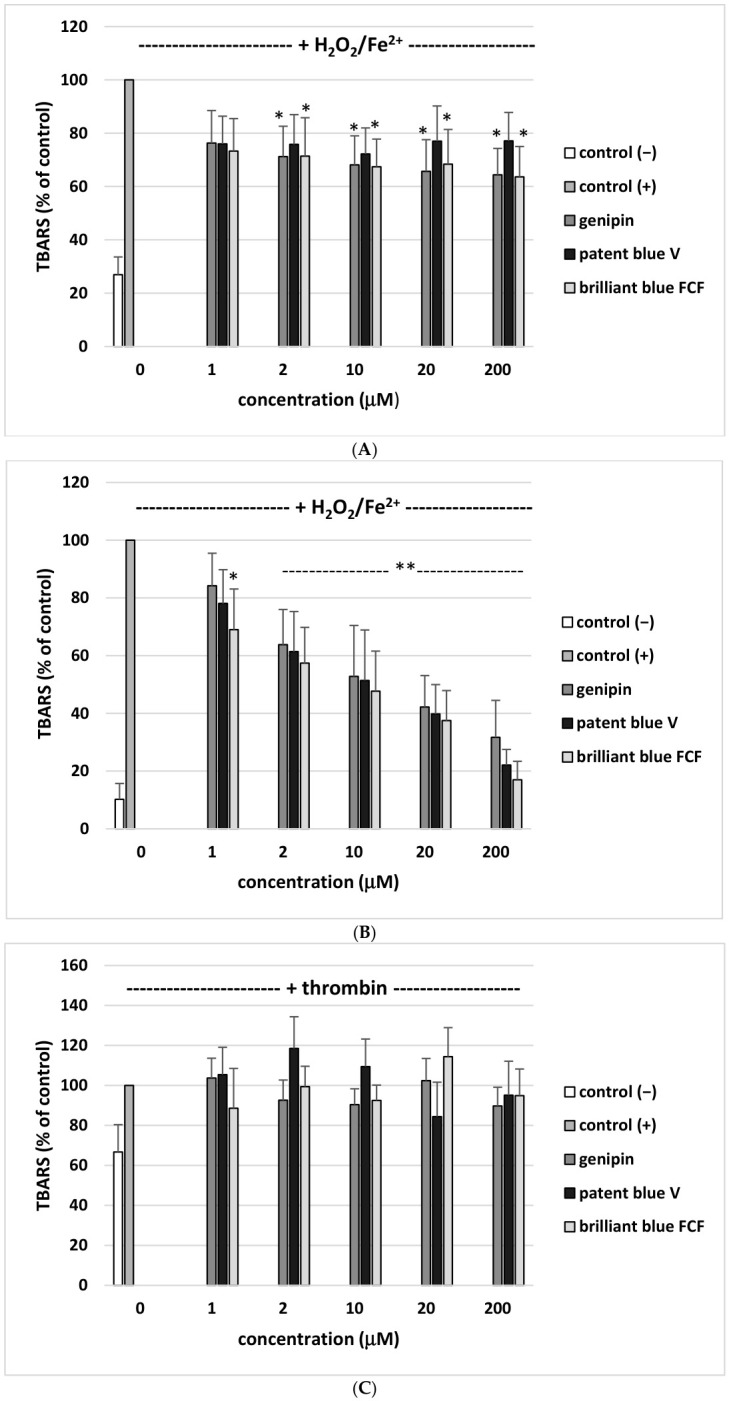
Effect of genipin, patent blue V, and brilliant blue FCF (at concentrations of 1, 2, 10, 20, and 200 μM) on lipid peroxidation (measured by level of TBARS) in plasma treated with H_2_O_2_/Fe^2+^ (**A**), in blood platelets treated with H_2_O_2_/Fe^2+^ (**B**), and in blood platelets treated with thrombin (**C**). Negative control (control (−)) refers to plasma/blood platelets not treated with H_2_O_2_/Fe^2+^, whereas positive control (control (+)) refers to plasma/blood platelets treated with H_2_O_2_/Fe^2+^ (**A**,**B**). Negative control (control (−)) also refers to blood platelets not treated with thrombin, whereas positive control (control (+)) also refers to blood platelets treated with thrombin (**C**). Differences between control (−) and control (+) were statistically significant (*p* < 0.01). Data represent means ± SD of 6 experiments; * *p* < 0.05, ** *p* < 0.01, *p* > 0.05 (compared with positive control).

**Figure 3:**
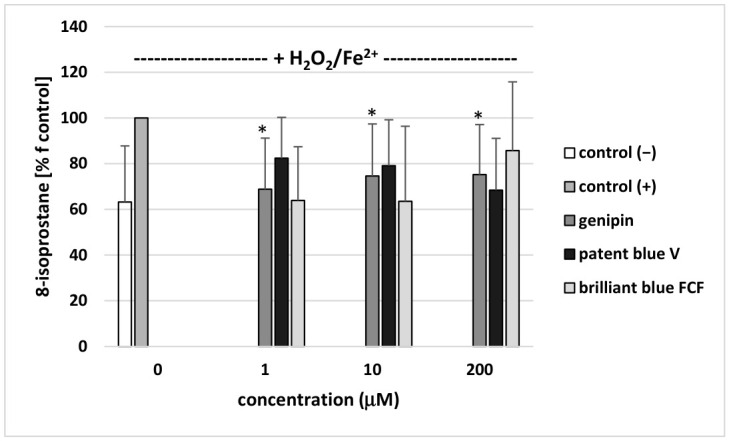
Effect of genipin, patent blue V, and brilliant blue FCF (at concentrations of 1, 10, and 200 μM) on lipid peroxidation (measured by level of 8-isoprostane) in plasma treated with H_2_O_2_/Fe^2+^. Negative control (control (−)) refers to plasma not treated with H_2_O_2_/Fe^2+^, whereas positive control (control (+)) refers to plasma treated with H_2_O_2_/Fe^2+^. Differences between control (−) and control (+) were statistically significant (*p* < 0.01). Data represent means ± SD of 3 experiments; * *p* < 0.05 (compared with positive control).

**Figure 4:**
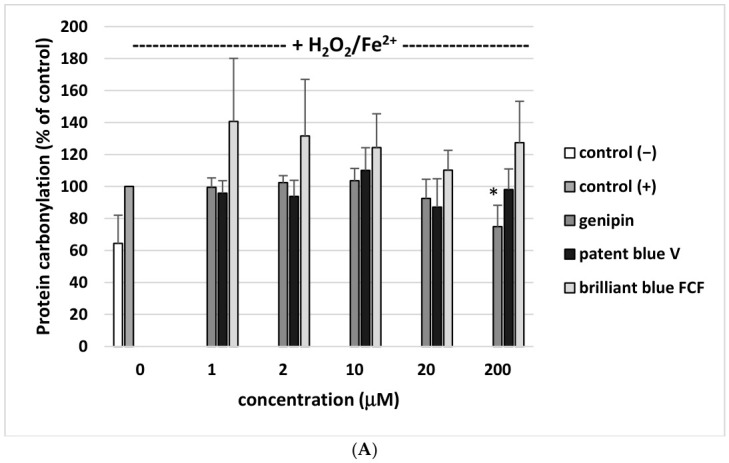

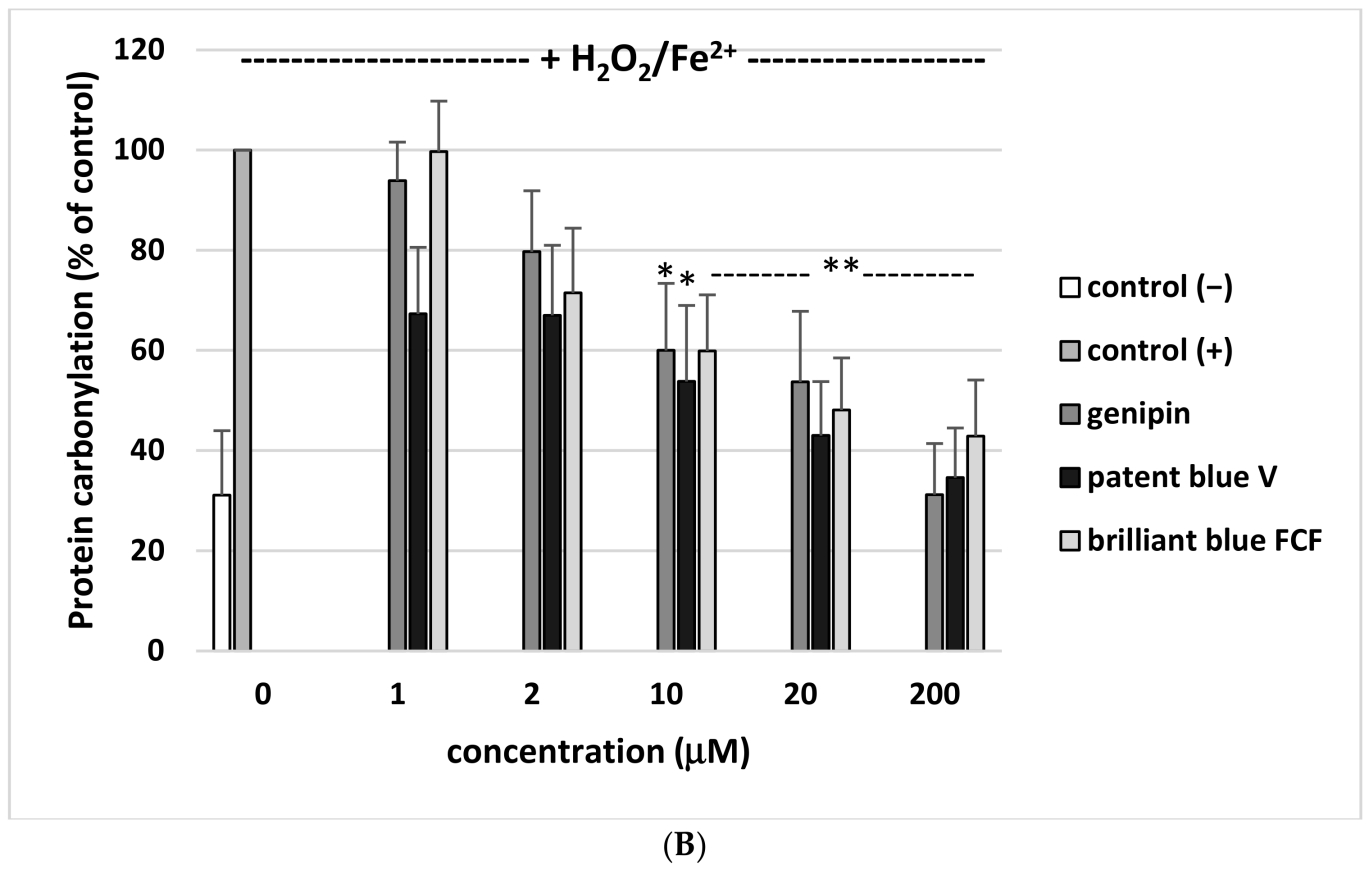
Effect of genipin, patent blue V, and brilliant blue FCF (at concentrations of 1, 2, 10, 20, and 200 μM) on protein carbonylation in plasma treated with H_2_O_2_/Fe^2+^ (**A**) and in blood platelets treated with H_2_O_2_/Fe^2+^ (**B**). Negative control (control (−)) refers to plasma/blood platelets not treated with H_2_O_2_/Fe^2+^, whereas positive control (control (+)) refers to plasma/blood platelets treated with H_2_O_2_/Fe^2+^ (**A**,**B**). Differences between control (−) and control (+) were statistically significant (*p* < 0.01). Data represent means ± SD of 6 experiments; * *p* < 0.05, ** *p* < 0.01, *p* > 0.05 (compared with positive control).

**Figure 5:**
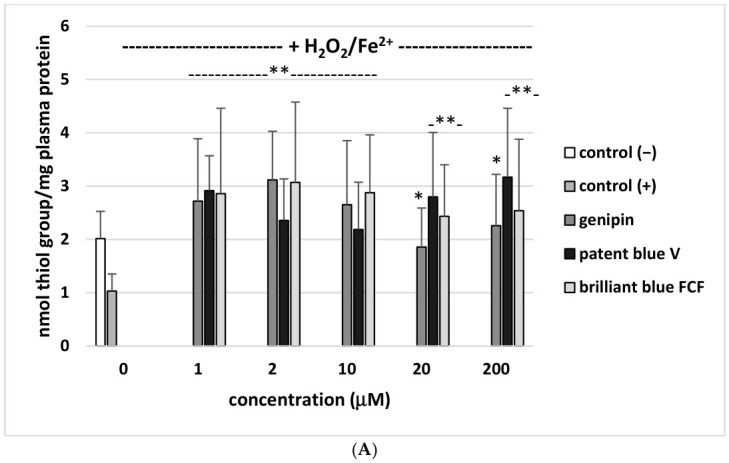

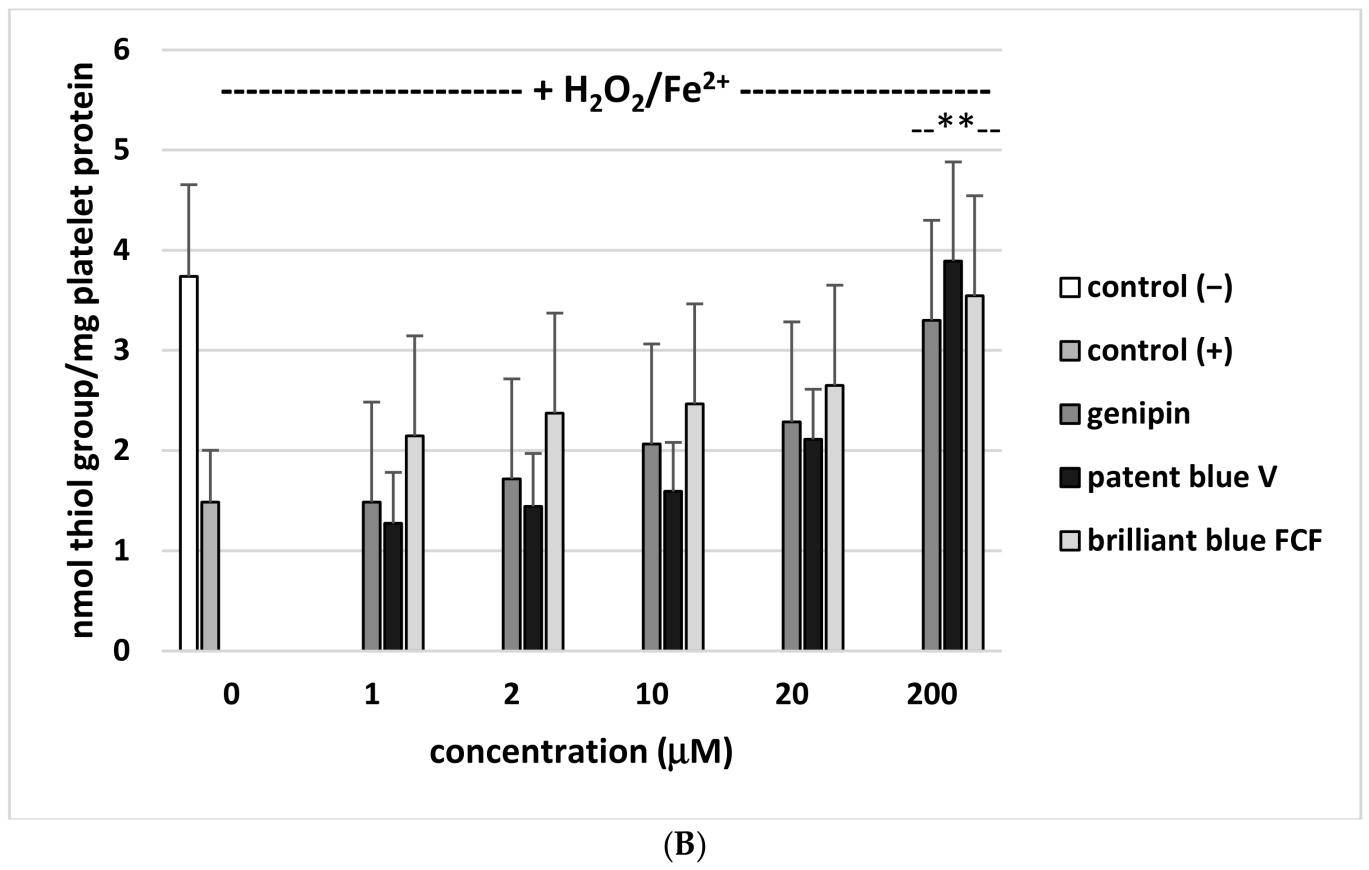
Effect of genipin, patent blue V, and brilliant blue FCF (at concentrations of 1, 2, 10, 20, and 200 μM) on level of thiol groups in plasma treated with H_2_O_2_/Fe^2+^ (**A**) and in blood platelets treated with H_2_O_2_/Fe^2+^ (**B**). Negative control (control (−)) refers to plasma/blood platelets not treated with H_2_O_2_/Fe^2+^, whereas positive control (control (+)) refers to plasma/blood platelets treated with H_2_O_2_/Fe^2+^ (**A**,**B**). Differences between control (−) and control (+) were statistically significant (*p* < 0.01). Data represent means ± SD of 6 experiments; * *p* < 0.05, ** *p* < 0.01, *p* > 0.05 (compared with positive control).

**Figure 6:**
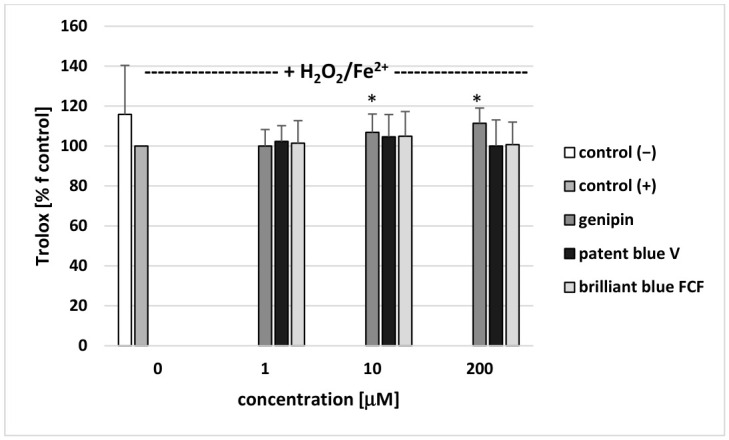
Effect of genipin, patent blue V, and brilliant blue FCF (at concentrations of 1, 10, and 200 μM) on total non-enzymatic antioxidant status of lipid peroxidation (measured by level of Trolox) in plasma treated with H_2_O_2_/Fe^2+^. Negative control (control (−)) refers to plasma not treated with H_2_O_2_/Fe^2+^, whereas positive control (control (+)) refers to plasma treated with H_2_O_2_/Fe^2+^. Differences between control (−) and control (+) were statistically significant (*p* < 0.01). Data represent means ± SD of 3 experiments; * *p* < 0.05 (compared with positive control).

Exposure of human plasma and human blood platelets to a strong oxidant—H_2_O_2_/Fe^2+^ (the donor of hydroxyl radicals)—resulted in enhanced levels of lipid peroxidation, carbonyl groups, and oxidation of protein thiols (Figure 2, Figure 3 and Figure 4). The antioxidant potential of genipin, brilliant blue FCF, and patent blue V human plasma and blood platelets (in vitro) is demonstrated in Figure 2, Figure 3, Figure 4, Figure 5 and Figure 6.

As demonstrated in Figure 2A, two tested blue colorants—genipin, and brilliant blue FCF (at four used concentrations: 2, 10, 20, and 200 µM)—reduced plasma lipid peroxidation induced by H_2_O_2_/Fe^2+^. Moreover, all tested blue colorants (genipin, brilliant blue FCF, and patent blue V; at the concentrations 2, 10, 20, and 200 µM) inhibited lipid peroxidation in blood platelets treated with H_2_O_2_/Fe^2+^ (Figure 2B). As demonstrated in Figure 2B, all tested blue colorants at the highest used concentration—200 µM—reduced lipid peroxidation in platelets stimulated by H_2_O_2_/Fe^2+^ by approximately 65–85% (for genipin—about 65%—for brilliant blue FCF—about 82%—and for patent blue V—about 77%) compared to blood platelets treated with only H_2_O_2_/Fe^2+^. The analysis of lipid peroxidation in blood platelets treated with thrombin showed that all tested blue colorants (concentration range: 1–200 µM) did not affect the level of TBARS (the marker of arachidonic acid metabolism) (Figure 2C). None of the tested synthetic blue colorants (1, 10, and 200 µM) changed the level of 8-isoprostane in human plasma treated with H_2_O_2_/Fe^2+^ (Figure 3). However, genipin (at three used concentrations: 1, 10, and 200 µM) statistically significantly decreased the level of 8-isoprostane (Figure 3).

We observed that only genipin (at the highest used concentration—200 µM) statistically significantly reduced plasma protein carbonylation induced by H_2_O_2_/Fe^2+^ (inhibition of this process: about 25%) (Figure 4A). All tested food colorants also decreased blood platelet protein carbonylation stimulated by H_2_O_2_/Fe^2+^, but their action was not always statistically significant. For example, genipin, patent blue V, and brilliant blue FCF (at the highest used concentration—200 µM) significantly inhibited this process by about 31.2%, 34.6%, and 42.9%, respectively (Figure 4B).

Moreover, we noted that all used blue food colorants (1–200 µM) had a protector effect on the change in the level of thiol groups in plasma proteins stimulated by H_2_O_2_/Fe^2+^, but this effect was not dose-dependent (Figure 5A), whereas only the tested colorants (at the highest used concentration—200 µM) changed the level of thiol groups in blood platelets treated with H_2_O_2_/Fe^2+^ (Figure 5B).

We found that none of the tested synthetic blue colorants (1, 10, and 200 µM) changed the level of Trolox in human plasma treated with H_2_O_2_/Fe^2+^ (Figure 5), whereas genipin (at two tested concentrations: 10 and 200 µM) statistically significantly decreased its level (Figure 6).

The effect of the tested blue food colorants (at one selected concentration—10 µM) are compared with ascorbic acid as a positive control (10 µM) in Table 1. Ascorbic acid demonstrated antioxidant activity not only in plasma but also in blood platelets. Stronger antioxidant properties were demonstrated by genipin (for six used models) than those demonstrated by Brillant blue FCF (for four used models) and patent blue V (for three used models).

We have also noted that all used blue colorants (1–200 µM) do not change the level of parameters of oxidative stress in plasma and blood platelets without H_2_O_2_/Fe^2+^.

## 3. Discussion

According to traditional Chinese medicine, herbs need to be processed in order to improve the efficacy and reduce the toxicity to humans. Fruits of *G. jasminosis* are broadly applied in clinical use and food additives (as a colorant), especially in China and other Asian countries. Results of Suzuki et al. [11] indicate that geniposide (the most representative iridoid glycoside in *G. jasminosis* fruits) has an antithrombotic action in vivo. Moreover, they suggest that inhibition of phospholipase A_2_ by this compound may be one possible antiplatelet mechanism. A few papers also indicate that genipin can modulate blood platelet activation. For example, Suzuki et al. [11] noted that this colorant inhibits mouse platelet aggregation—stimulated by collagen (in vitro). Our earlier results also demonstrated that genipin has anti-adhesive activity without the cytotoxicity against blood platelets [7].

Blood platelet activation is associated with arachidonic acid metabolism, in which different intermediate products (including thromboxane A_2_) are synthetized. In our present experiment, the TBARS concentration was used as an indicator of this process in human blood platelets stimulated by a strong physiological agonist—thrombin. For the first time, our present data, derived using washed platelets, indicates that genipin (1–200 µM) has no effect on the arachidonic acid pathway. Based on these results, we suggest that the antiplatelet action of genipin might be due to other mechanisms. For example, it may inhibit platelet activation by decreasing the binding between various receptors and platelet agonists. Therefore, more research is needed to better understand its antiplatelet mechanism of action.

Food products include not only natural blue colorants but also often synthetic colorants, including brilliant blue FCF, which is added to soft drinks, liquors, chewing gums, candies, jellies, and dairy products. Another synthetic blue colorant—patent blue V—is also used as a colorant in various food products, including dried fruits, cheeses, jellies, liqueurs, and sauces [12,13,14,15,16]. Our preliminary results demonstrated that blue synthetic colorants, especially patent blue V, have procoagulant properties as well as cytotoxic activity—they damage blood platelets in vitro (measured based on extracellular lactate dehydrogenase) [7]. An important, novel aspect of our present finding is that the tested synthetic colorants (brilliant blue FCF and patent blue V) do not affect arachidonic acid metabolism (in vitro models).

The biological properties of blue food colorants at the molecular level remain only partially understood. In addition, scientific research on their pro- and antioxidant activity is still limited. So far, there has been no data on the anti- or prooxidant properties of genipin or other blue food colorants in various elements of hemostasis in the presence of hydroxyl radicals, which are generated by the reaction of H_2_O_2_ and transition metal ions and are especially reactive and damaging. The model of oxidative stress stimulated by the H_2_O_2_/Fe^2+^ system is very often used, but various elements of blood, especially plasma, are complex biological mixtures containing not only metal-binding proteins but also endogenous antioxidants, which may influence the generation of hydroxyl radicals during the Fenton reaction. In this study, we found that exposure of plasma and blood platelets to H_2_O_2_/Fe^2+^ resulted in a significantly enhanced level of various biomarkers of oxidative stress, including oxidation of thiols, lipid peroxidation, and others. Therefore, the present in vitro study examines the pro- or antioxidant activity of three selected blue food colorants (one natural colorant—genipin—and two synthetic colorants—brilliant blue FCF and patent blue V) in selected elements of the cardiovascular system treated with the donor of hydroxyl radicals—H_2_O_2_/Fe^2+^. We aim to also valorize their potential as new bioactive antioxidants and to evaluate their health-promoting value in the cardiovascular system context. The results reflect the effect of blue food colorants not only on oxidative damage but also on other biological materials. Based on our obtained results, for the first time, we have noted that all tested blue food colorants (depending on the dosage) can modulate oxidative stress stimulated by H_2_O_2_/Fe^2+^ in in vitro models. The antioxidant mechanisms of tested blue food colorants may include scavenging oxidants. It is also worth emphasizing that brilliant blue FCF reduced lipid peroxidation (measured by the level of TBARS) in plasma treated with H_2_O_2_/Fe^2+^, but this colorant did not inhibit the oxidation of ABTS. Therefore, we suggest that the mechanism responsible for its antioxidant activity might be linked to the activity of antioxidant enzymes, including glutathione peroxidase, catalase, superoxide dismutase, and others.

All the tested compounds differed in terms of their antioxidant properties, and these differences may be associated with their different chemical structures. These results are consistent with other studies on various chemical compounds present in foods, including alkaloids, phenolic compounds and food colorants, which may exert inhibitory or stimulatory effects on oxidative stress, depending on their concentration and bioavailability [16,17,18].

In addition, results of Yang et al. [19] indicate that various processing methods may influence the pharmacokinetics of genipin. Genipin could be detected in rat plasma after administration of crude extract from *G*. *jasminosis* fruits, but it was not detected in plasma after administration of the ginger mix-frying *G*. *jasminosis* extract. Moreover, genipin is absorbed via the intestine and transported to the liver through the portal blood stream. Synthetic blue colorants, such as patent blue V and brilliant blue FCF, are poorly absorbed from the gastrointestinal tract [8,20].

The results of Xia et al. [21] demonstrated that genipin (50 µ/mL) stimulates toxicity through oxidative stress and apoptosis in zebrafish. The increase in oxidative stress induced by genipin triggered the generation of ROS and TBARS and decreased the activity of superoxide dismutase. After genipin treatment, the suppression of the antioxidant capacity was also observed. However, Zhao et al. [22] noted that genipin (5–100 µM) protects against injury stimulated by H_2_O_2_ (5–100 µM) in retinal pigment epithelial cells. Another study also indicates that this natural blue colorant attenuated cisplatin-stimulated nephrotoxicity by abrogating oxidative stress in a murine model [23]. In addition, genipin reduced oxidative damage in osteoarthritis by inhibition of phosphoglycerate kinase 1 in vitro [24]. Deng et al. [25] constructed a model of H_2_O_2_-stimulated oxidative stress in human periodontal ligament cells treated with genipin, and they observed that genipin regulates oxidative damages by maintaining mitochondrial homeostasis, promoting glucose transporter expression, and enhancing glucose uptake.

Here, we have noted that genipin (at two used concentrations: 10 and 200 µM) induces statistically significant changes in the level of Trolox in human plasma treated with H_2_O_2_/Fe^2+^.

Gorczyca et al. [26] have characterized biological properties of genipin-crosslinked porous chitosan–collagen–gelatin scaffolds using chitosan–CO_2_ solution. They observed good antioxidant properties (ABTS assay) and especially very low in vitro cytotoxicity against fibroblasts. In the other experiment, genipin-crosslinked gelatin/chitosan-based functional films incorporated with rosemary essential oil and quercetin were used, but crosslinking by genipin did not significantly affect the antioxidant performance of the film. In contrast, the addition of the functional fillers (quercetin and rosemary essential oil) significantly increased the antioxidant activity of the film [27].

Recently, results of Angi et al. [28] have demonstrated that a genipin derivative—mito-genipin—also modulates oxidative stress in macrophages. For example, mito-genipin inhibited ROS production. Other genipin derivatives (0.01 µM) have also effectively attenuated glutamate-induced oxidative damage (by inhibiting ROS over-accumulation and reducing TBARS content) in HT22 cells [29]. It is important that genipin (25 mg/kg/day, for 12 days) ameliorates hepatic oxidative stress in rats [30]. Neri-Numa et al. [31] demonstrated that genipap fruit extract (60.8 mg g fwd.) has antioxidant action in various tumor cell lines in vitro.

For the first time, the action of both a natural colorant—genipin—and synthetic colorants (brilliant blue FCF and patent blue V) on selected biomarkers of oxidative stress was compared with that of ascorbic acid. However, ascorbic acid (10 µM) had better antioxidant properties than the three tested blue food colorants when we measured its effect on the level of oxidative stress in blood platelets and plasma. Another important aspect of our finding is that genipin (10 µM) and ascorbic acid (10 µM) had similar inhibitory effects on the level of TBARS in plasma treated with H_2_O_2_/Fe^2+^ and the level of carbonyl groups in platelet proteins treated with H_2_O_2_/Fe^2+^. In addition, genipin (10 µM) was more effective at preventing thiol group oxidation than ascorbic acid (10 µM) in plasma treated with H_2_O_2_/Fe^2+^.

Oxidative stress damages various components of blood by generating ROS, which induce lipid peroxidation, leading to structural impairment and the formation of harmful products, such as not only aldehydes but also isoprostanes. At the same time, oxidative stress depletes the plasma’s non-enzymatic antioxidant capacity. In our present experiments, ascorbic acid was more effective at preventing lipid peroxidation (measured by 8-isoprostane) and the plasma Total Non-Enzymatic Antioxidant Capacity (NEAC) than genipin, but its effect was statistically significant. The NEAC measures the collective ability of low-molecular-weight substances, including albumin and vitamins C and E, demonstrating a key indicator of antioxidant status. It is important that the NEAC is commonly used to screen the level of oxidative stress and reflects the ability to combat oxidative damages, unlike enzymatic antioxidants. However, the use of hydroxyl radicals as the oxidant is not recommended in view of the high and non-specific reactivity of this species [32]. Therefore, more research is needed to better understand the antioxidant mechanism of various blue food colorants, especially genipin, in human plasma treated with other oxidants (for example, peroxynitrite).

In conclusion, the present study provides the first data on the antioxidant potential of genipin, brilliant blue FCF, and patent blue V in selected elements of blood treated with H_2_O_2_/Fe^2+^. Earlier and current studies have indicated the promising potential of these blue food colorants, especially genipin (without cytotoxicity toward human blood platelets), which can modify the oxidative stress of platelets and plasma in vitro at concentrations (1–200 µM) which can be obtained in blood during its administration. However, the presented results have limitations, especially regarding the mechanistic clarity surrounding the antioxidant properties of the tested blue food colorants. Therefore, further in vivo experiments, including metabolic transformations of food colorants, their distribution, and their interaction with endogenous redox systems, are needed to provide a better understanding of their antioxidant potential.

## 4. Materials and Methods

### 4.1. Chemical Reagents

Dimethylsulfoxide (DMSO), trichloroacetic acid (TCA), thiobarbituric acid (TBA), Phosphate-Buffered Saline (PBS), hydrochloric acid (HCl), 5,5′-Dithiobis(2-nitrobenzoic acid), commonly known as Ellman’s reagent or DTNB, and guanidine hydrochloride (GdnHCl) were purchased from Sigma-Aldrich (St. Louis, MO, USA). 2,4-dinitrophenylhydrazine (DNPH) and H_2_O_2_ were purchased from Stanlab Sp. J. (Lublin, Poland). Brilliant blue FCF was purchased from Warchem (Zakręt, Poland). Genipin and patent blue V were purchased from Pol-Aura (Morąg, Poland). Sodium chloride (NaCl), ethylenediaminetetraacetic acid (EDTA), and sodium dodecyl sulfate (SDS) were purchased from POCH (Avantor performance materials, Gliwice, Poland). Other reagents were obtained from commercial distributors and were of the highest grade available.

### 4.2. Preparation of Stock Solution of Tested Colorants

Genipin, patent blue V, and brilliant blue FCF were dissolved in water. The final concentrations of the tested blue colorants in human plasma and blood platelet samples were 1, 2, 10, 20, and 200 μM.

### 4.3. Plasma and Blood Platelet Samples

Human whole blood was drawn from volunteers at the “Diagnostyka” blood collection center (Lodz, Poland). All volunteers (aged 21–25; *n* = 6 (3 men and 3 women)) were healthy and did not smoke. The donors did not drink alcohol or take medicine (including antiplatelet drugs, for example, aspirin or its derivatives) for two weeks before blood collection. Informed consent was obtained from the participants one day prior to blood collection. The anticoagulant was citrate/phosphate/dextrose/adenine (CPDA).

All procedures were performed according to the guidelines of the Helsinki Declaration for Human Research. The research was conducted with the consent of the Bioethics Committee at the University of Łódź (2/KBBN-UŁ/III/2014).

Plasma was obtained from whole blood by differential centrifugation (2800× *g*, 20 min, room temperature). The isolation of blood platelets from whole blood (by differential centrifugation) has been described previously [33]. The platelet count was measured by a spectrophotometric measurement with a UV–Visible Helios-α at 800 nm. Then, the platelets were diluted to the level of 2.0 × 10^8^/mL with Barber’s buffer (a modified Tyrode’s buffer: 0.14 M NaCl; 0.014 M Tris; 5 mM glucose; pH 7.4).

In all experiments, plasma or blood platelets were incubated for 30 min at 37 °C with blue colorants (at final concentrations of 1–200 µM).

### 4.4. Lipid Peroxidation Measurement

Lipid peroxidation was quantified by measuring the concentration of thiobarbituric acid reactive substances (TBARS), according to the method described by Bartosz [34]. The TBARS concentration was calculated using the molar extinction coefficient (ε = 156,000 M^−1^ cm^−1^) and was expressed as nmol/mL of plasma or as nmol/mL of blood platelets.

### 4.5. Carbonyl Group Measurement

The carbonyl groups were determined in plasma protein according to Levine et al. [35] and Bartosz [34]. The carbonyl group concentration was calculated using a molar extinction coefficient (ε = 22,000 M^−1^ cm^−1^) and was expressed as nmol/mg of plasma protein or as nmol/mg blood platelet protein.

### 4.6. Thiol Group Measurement

The thiol group content was measured spectrophotometrically (the absorbance at 412 nm) using the SPECTROstar Nano Microplate Reader (BMG LABTECH, Ortenberg, Germany), with Ellman’s reagent—5,5′-dithio-bis-(2-nitrobenzoic acid)—according to the method described by Bartosz [34]. The thiol group concentration was calculated using a molar extinction coefficient (ε = 13,600 M^−1^ cm^−1^) and was expressed as nmol/mg of plasma protein or as nmol/mg of blood platelet protein.

### 4.7. Free 8-Isoprostane in Plasma

The level of free 8-isoprostane in human plasma was determined with an 8-Isoprostane Express ELISA Kit (Item No. 516360, Cayman Chemical, Ann Arbor, MI, USA), and the concentration of free 8-isoprostane in the samples was calculated with a pre-configured analysis tool provided by the manufacturer. Its concentration was expressed as pg/mL of plasma.

### 4.8. Total Non-Enzymatic Antioxidant Status of Plasma

The total non-enzymatic antioxidant status was measured with an Antioxidant Assay Kit (Item No. 709001, Cayman Chemical). This assay is based on the ability of antioxidants to inhibit the oxidation of 2,2′-azino-bis(3-ethylbenzothiazoline-6-sulfonic acid) (ABTS) to ABTS^•+^ by metmyoglobin. The calculations were carried out with a pre-setup tool provided by the manufacturer.

### 4.9. Data Analysis

Statistical analyses were conducted using Statistica software version 10 (StatSoft 13.3, TIBCO Software Inc., Palo Alto, CA, USA). Data normality was evaluated using normal probability plots, while variance homogeneity was assessed by the Brown–Forsythe test. Differences among and between experimental groups were analyzed using one-way analysis of variance (ANOVA), followed by Duncan’s multiple comparison test. For clarity, only statistically significant differences between the tested compounds and the control or positive control groups were indicated.

## Figures and Tables

**Table 1:** A comparison of the antioxidant activities of genipin, patent blue V, brilliant blue FCF, and ascorbic acid (10 µM) in human plasma and blood platelets treated with H_2_O_2_/Fe^2+^ (in vitro).

Chemical Compounds	Biomarkers of Oxidative Stress							
	TBARS (% of Control)		8-Isoprostane (% of Control)	Protein Carbonylation (% of Control)		Oxidation of Protein Thiols (% of Control)		Trolox (% of Control)
	Plasma	Blood Platelets	Plasma	Plasma	Blood Platelets	Plasma	Blood Platelets	Plasma
Control	100	100	100	100	100	100	100	100
Genipin	68.7 ± 10.9 (*p* < 0.05)	52.8 ± 17.7 (*p* < 0.01)	74.6 ± 22.8 (*p* < 0.05)	103.6 ± 7.7 (*p* > 0.05)	53.7 ± 14.1 (*p* < 0.02)	261.8 ± 59.1 (*p* < 0.01)	173.5 ± 34.2 (*p* > 0.05)	106.8 ± 9.2 (*p* < 0.05)
Patent blue V	72.2 ± 23.2 (*p* > 0.05)	51.4 ± 17.5 (*p* < 0.01)	79.1 ± 20.1 (*p* > 0.05)	110.1 ± 14.2 (*p* > 0.05)	43.0 ± 10.8 (*p* < 0.01)	228.9 ± 67.9 (*p* < 0.05)	178.9 ± 44.1 (*p* > 0.05)	104.6 ± 11 (*p* > 0.05)
Brilliant blue FCF	67.4 ± 10.4 (*p* < 0.05)	47.7 ± 13.8 (*p* < 0.01)	63.5 ± 32.9 (*p* > 0.05)	124.3 ± 21.2 (*p* > 0.05)	48.1 ± 10.4 (*p* < 0.01)	255.2 ± 69.7 (*p* < 0.05)	135.6 ± 29.9 (*p* > 0.05)	104.9 ± 12.4 (*p* > 0.05)
Ascorbic acid	68.2 ± 12.1 (*p* < 0.05)	69.1 ± 12.4 (*p* < 0.05)	56.9 ± 13.3 (*p* < 0.01)	42.1 ± 9.8 (*p* < 0.05)	58.1 ± 12.2 (*p* < 0.02)	148.2 ± 19.2 (*p* < 0.05)	166.8 ± 22.1 (*p* < 0.05)	121.9 ± 9.3 (*p* < 0.05)

## Data Availability

The raw data supporting the conclusions of this article will be made available by the authors upon request.

## Abbreviations

CPDA—citrate/phosphate/dextrose/adenine; CVDs—cardiovascular diseases; DMSO—dimethylsulfoxide; PBS—phosphate-buffered saline; ROS—reactive oxygen species; TBA—thiobarbituric acid; TBARS—thiobarbituric acid reactive substances.
